# Zr-Based Metal−Organic Frameworks with Phosphoric Acids for the Photo-Oxidation of Sulfides

**DOI:** 10.3390/ijms232416121

**Published:** 2022-12-17

**Authors:** Zhenghua Zhao, Mingjie Liu, Kai Zhou, Hantao Gong, Yajing Shen, Zongbi Bao, Qiwei Yang, Qilong Ren, Zhiguo Zhang

**Affiliations:** 1 Key Laboratory of Biomass Chemical Engineering of Ministry of Education, College of Chemical and Biological Engineering, Zhejiang University, Hangzhou 310058, China; 2 Institute of Zhejiang University—Quzhou, Quzhou 324000, China

**Keywords:** metal−organic frameworks, phosphoric acid, photo-oxidation, oxygen radical

## Abstract

Heterogeneous Brønsted acidic catalysts such as phosphoric acids are the conventional activators for organic transformations. However, the photocatalytic performance of these catalysts is still rarely explored. Herein, a novel Zr-based metal−organic framework **Zr-MOF-P** with phosphoric acids as a heterogeneous photocatalyst has been fabricated, which shows high selectivity and reactivity towards the photo-oxidation of sulfides under white light illumination. A mechanism study indicates that the selective oxygenation of sulfides occurs with triplet oxygen rather than common reactive oxygen species (ROS). When **Zr-MOF-P** is irradiated, the hydroxyl group of phosphoric acid is converted into oxygen radical, which takes an electron from the sulfides, and then the activated substrates react with the triplet oxygen to form sulfoxides, avoiding the destruction of the catalysts and endowing the reaction with high substrate compatibility and fine recyclability.

## 1. Introduction

Porous solid Brønsted acids such as phosphoric acids are important heterogeneous catalysts for diverse chemical reactions [1,2,3]. An effective way to construct these catalysts is to introduce them into highly porous and stable metal−organic frameworks (MOFs) [4,5,6]. Phosphoric acid-based MOFs have received extensive attention in recent years due to their high reactivity and selectivity towards many types of reactions, especially asymmetric transformations [7,8,9,10,11]. Diverse phosphoric acid ligands are synthesized to construct different MOFs; among these, the coordination of carboxyl groups with Zr(IV) show the best stability [10,11]. However, in view of the large conjugate structure associated with phosphoric acid-based MOFs, their photocatalytic performance is rarely explored.

MOFs with tailorable structures and high porosities have been demonstrated to be efficient photocatalysts towards various types of reactions, such as H_2_O splitting [12,13,14,15], CO_2_ reduction [16,17,18,19], organic pollutant degradation [20,21,22,23], and organic transformations [24,25,26,27,28]. Ligand structure in MOFs is essential to the regulation of their photocatalytic performance, including the photoresponsivity and active site. Compared to other ligands, phosphoric acids have versatile sites towards different substrates [29,30], and the moderate acidity of phosphoric acid makes it an ideal candidate for the construction of materials to undergo excited-state proton transfer (ESIPT) under photo-excitation, which is a common reaction in molecules with acidic hydrogen atoms and conjugate systems [31,32,33]. ESIPT products are reactive and the reaction is in general wholly reversible, so it would be an effective strategy to construct novel photocatalytic MOFs through introducing phosphoric acids into photo responsive ligands. Thereinto, binaphthol (BINOL)-based phosphoric acids with a large conjugated structure, ESIPT propriety, fine visible-light-response, and good modifiability could be functioned as effective photocatalysts [34,35,36,37,38,39]. In this context, BINOL is chosen as a potential skeleton to fabricate photo responsive phosphoric acid ligands for MOF construction.

Photocatalyzed selective oxidation of sulfides using oxygen as the oxidizer is an environment-friendly way to produce sulfoxides, which are key intermediates for bioactive ingredients in the pharmaceutical industry [40,41,42]. Generally, oxygen is activated by the photocatalyst through energy transfer or electron transfer to generate reactive oxygen species (ROS) such as ^1^O_2_ and O_2_^−•^, followed by the reaction with sulfide to produce sulfoxide [43,44,45,46,47,48]. Due to the high activity of ROS, the generated sulfoxide and some substituent groups with low stability would also be oxidized, causing low selectivity and poor substrate compatibility. Moreover, the photocatalysts might also be affected by hyperactive ROS and the lifetime of catalysts is shortened, which are unfavorable for the application of photocatalysts, especially the recycling of heterogeneous photocatalysts. Avoiding the production of ROS, making the photocatalyst directly activate the sulfide and react with triplet oxygen, is an effective way to improve the selectivity and substrate compatibility [49]. Considering that the phosphoric acid-based materials may follow the ESIPT route, we believe phosphoric acids containing Zr-based MOFs would be a good candidate material for the selective photocatalytic oxygenation of sulfides to sulfoxides and may have better selectivity and substrate compatibility. In this view, a BINOL-based phosphoric acid ligand and corresponding Zr-MOFs were fabricated and employed in this reaction; their catalytic performance and reaction mechanism were also investigated.

## 2. Results and Discussion

### 2.1. Synthesis and Characterization

**Scheme 1:**
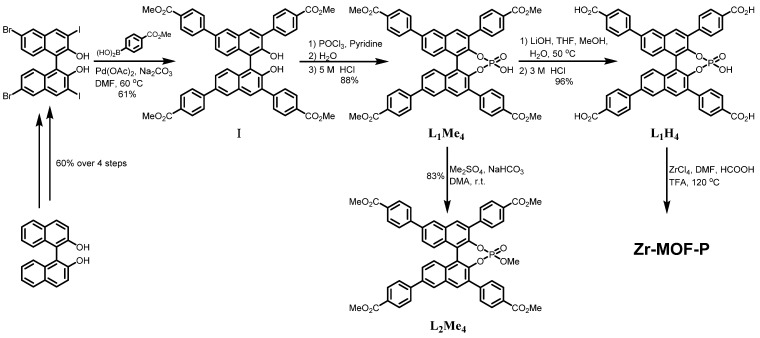
Synthesis of ligand and **Zr-MOF-P**.

The ligand 3,3′,6,6′-tetrakis(4-benzoic acid)-1,1′-binaphthyl phosphate (**L_1_H_4_**) was synthesized with an optimized route according to the literature [7]; the new route was shortened to seven steps and the total yield was increased to 31%. To study the effect of the phosphate hydroxyl group, a phosphate-hydroxyl-protected ligand 3,3′,6,6′-tetrakis(4-methyl benzoate)-1,1′-binaphthyl methyl phosphate (**L_2_Me_4_**) was also synthesized by an additional methylation reaction for the precursor of **L_1_H_4_** (**L_1_Me_4_**). **Zr-MOF-P** was prepared through a solvothermal reaction with **L_1_H_4_**, ZrCl_4_, formic acid, and trifluoroacetic acid (TFA) in *N*,*N*-dimethylformamide (DMF) at 120 °C as light yellow octahedral crystals (Scheme 1).

**Figure 1:**
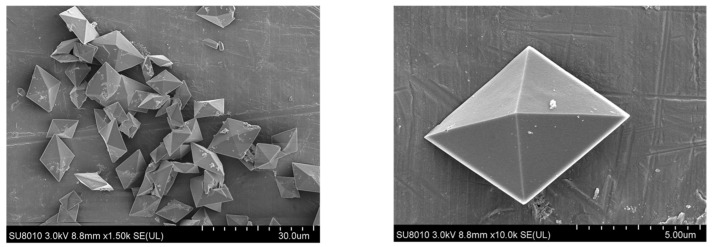
SEM images of the synthesized **Zr-MOF-P**.

**Figure 2:**
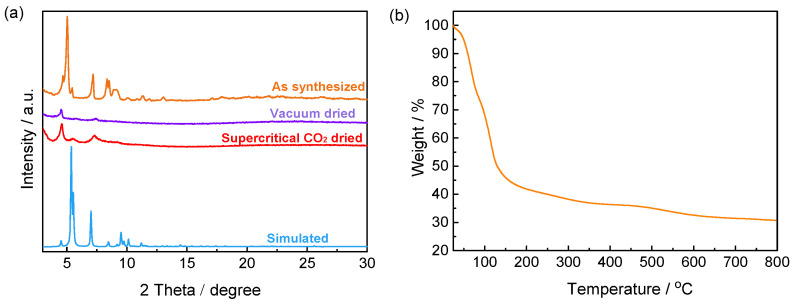
(**a**) PXRD patterns of **Zr-MOF-P** under different conditions. (**b**) TGA profile of **Zr-MOF-P**.

**Figure 3:**
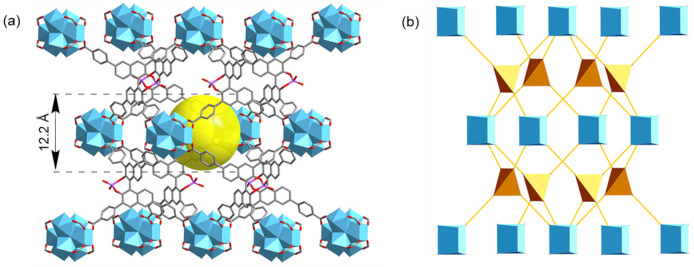
(**a**) Structure of **Zr-MOF-P**. C, gray; O, red; P, pink; Zr, cyan polyhedra; the yellow sphere is the cavity; H atoms are omitted for clarity. (**b**) The **flu** topology of **Zr-MOF-P**. The orange and cyan polyhedra represent 4- and 8-connected nodes, respectively.

Scanning electron microscope (SEM) images (Figure 1) and powder X-ray diffraction (PXRD) patterns (Figure 2a) confirmed that **Zr-MOF-P** was successfully synthesized. Single crystal X-ray diffraction showed that **Zr-MOF-P** crystallizes in the monoclinic space group (a = 21.112 Å, b = 38.991 Å, c = 19.209 Å, and β = 120.902°) and is similar to most of the reported Zr-MOFs composed of carboxylate-based tetrahedral linkers [50,51,52,53]; it also exhibited the **flu** topology (Figure 3, Table S1, and Figure S1). The phosphoric acid ligand presents a distorted tetrahedron structure, with a 55.70° dihedral angle between the two naphthalene groups, constructing a cavity with a diameter of 12.2 Å. The total solvent accessible volume of **Zr-MOF-P** is estimated to be 73.6%, calculated by the PLATON routine [54]. The simulated PXRD pattern is similar to the experimental data, demonstrating the phase purity of **Zr-MOF-P**. Due to the large ligand and high porosity of **Zr-MOF-P**, its crystallinity was destroyed after removing the solvent molecules in the channels by vacuum-drying. Therefore, we tried the supercritical CO_2_-drying method to keep its structural integrity, and the results showed that the crystal structure of the dried MOF was still partly destroyed (Figure 2a). Considering that the removal of the solvent would break the structure of **Zr-MOF-P**, it was directly used in the photocatalytic reactions after being washed with DMF several times through suction filtration. Unsurprisingly, **Zr-MOF-P** showed high thermal stability, thermogravimetric analysis (TGA) indicated that the guest molecules are removed before the temperature reaches 160 °C, and the frameworks remained stable below 300 °C (Figure 2b).

**Figure 4:**
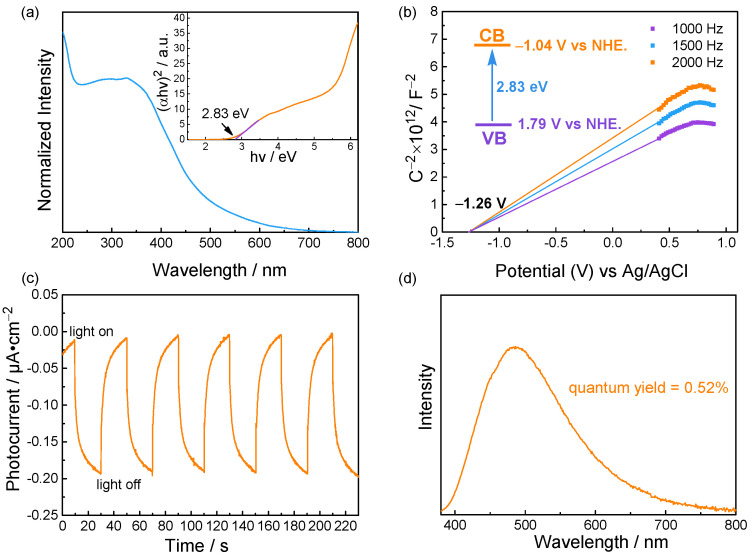
(**a**) UV–Vis diffuse reflectance spectrum of **Zr-MOF-P**; insets are the Tauc plots. (**b**) Mott–Schottky plots for **Zr-MOF-P**. (**c**) Photocurrent responses of **Zr-MOF-P**. (**d**) Fluorescence spectrum and quantum yield of **Zr-MOF-P**, λ_ex_ = 320 nm.

To identify the possible photocatalytic application of **Zr-MOF-P**, its photo-electrochemical properties were tested. As shown in Figure 4a, UV–Vis spectra indicated that this MOF could be excited by visible light for its obvious absorption below 600 nm, and the band gap was estimated to be 2.83 eV. Mott–Schottky measurements were performed at the frequency of 1000, 1500, and 2000 Hz to identify the semiconductor characteristics of **Zr-MOF-P**, the flat band position determined from the same intersection is about −1.26 V vs. Ag/AgCl (−1.04 V vs. NHE), and the positive slope of the C^–2^ values indicates the character of n-type semiconductors [55,56,57]. Thus, the conduction band (CB) is −1.04 V vs. NHE, and the valence band (VB) is 1.79 V vs. NHE (Figure 4b). The VB of **Zr-MOF-P** is higher than the oxidation potentials of sulfides, but lower than that of sulfoxides [49], indicating that it could be used in the photo-oxidation of sulfides to sulfoxides. Photo-electrochemical measurements showed that **Zr-MOF-P** had an obvious photocurrent response, illustrating that the hole−electron pair could be separated under visible light irradiation (Figure 4c). The weak fluorescence emission indicated the low electron−hole recombination rate in **Zr-MOF-P**, which favored the electron transfer between photocatalysts and substrates (Figure 4d). All the photo-electrochemical measurements clearly demonstrated that **Zr-MOF-P** would be an ideal photocatalyst for the photo-oxidation of sulfides to sulfoxides.

### 2.2. Photo-Oxidation of Thioanisol

Considering the excellent photo-electric performance of **Zr-MOF-P**, we investigated its photocatalytic activity towards the photo-oxidation of sulfide into sulfoxide, and thioanisole was selected as the model substrate. The reaction was initially carried out in acetonitrile with **Zr-MOF-P** under white light irradiation and an O_2_ atmosphere at room temperature. As shown in Table 1, after 9 h, 19% of sulfide was oxidized into sulfoxide and no overoxidized product (sulfone) was produced. Based on the reported works, the yield is obviously affected by the type of solvents. Therefore, different solvents were explored, and the protic solvent trifluoroethanol (TFEA) was found to be the optimal solvent with a yield of 97% (Table 1, entry 5). Comparative experiments indicated that the photocatalyst, white light irradiation, and O_2_ are all indispensable (Table 1, entries 8–10). Moreover, the reaction still went smoothly on the gram scale with a yield as high as 95%.

### 2.3. Photocatalytic Mechanism

Most of the research reported that ROS such as ^1^O_2_, O_2_^−•^, or ∙OH originating from oxygen under the activation of a photocatalyst were important active species in the photocatalytic oxidation of sulfide. Therefore, we carried out a series of experiments to confirm whether ROS participate in the **Zr-MOF-P**-catalyzed reaction. Quenching experiments through adding different scavengers of ROS was firstly performed. Diazabicyclo[2.2.2]octane (DABCO, TCI, Tokyo, Japan) is a scavenger for ^1^O_2_; its addition showed no effect on the yield of sulfoxide, excluding the participation of ^1^O_2_ in the reaction (Table 2, entry 2). O_2_^−•^ is another common ROS participating in the photocatalytic oxidation reaction, while the reaction still went smoothly with the addition of benzoquinone (BQ, J&K Scientific, Beijing, China) as an O_2_^−•^ scavenger (Table 2, entry 3). Other ROS such as ∙OH and H_2_O_2_ are also capable of oxidizing sulfide, which were excluded through the quenching experiments with the addition of *i*-PrOH and catalase (TCI, Tokyo, Japan), respectively (Table 2, entries 4 and 5). The radical scavenger hydroquinone (HQ, J&K Scientific, Beijing, China) and electro trapper CuSO_4_ showed significant inhibition to the reaction, demonstrating that the reaction might have undergone an electron-transfer-induced free radical pathway (Table 2, entries 6 and 7). Moreover, the addition of the sulfide radical cation scavenger 1,4-dimethoxybenzene (DMB, Aladdin, Shanghai, China) also repressed the reaction with a decreased yield of 78% (Table 2, entry 8). Considering that the optimal solvent TFEA is conducive to maintaining the stability of cations [58,59], the sulfide radical cation should be a critical intermediate in the catalytic process. Quenching experiments using **L_1_Me_4_** as the catalyst also showed similar results (Table 2, entries 9–15), which indicated that the ligand in **Zr-MOF-P** was the active component.

**Figure 5:**
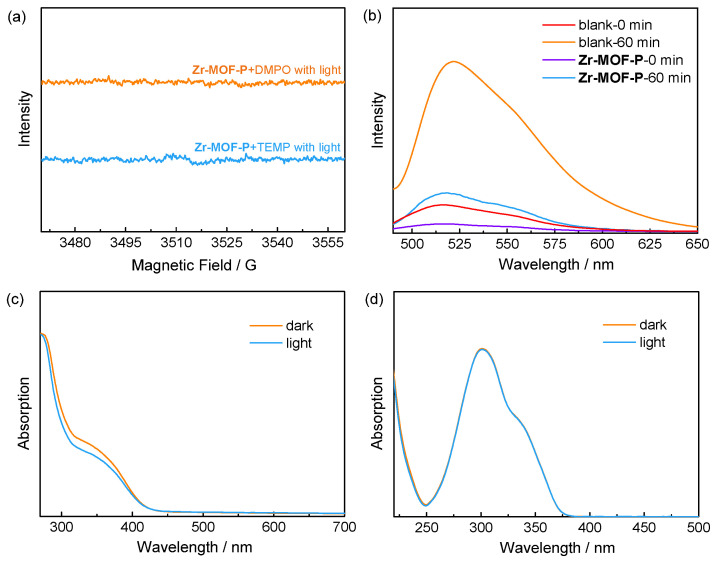
(**a**) EPR spectra of **Zr-MOF-P** using TEMP and DMPO as trapping agents under irradiation. (**b**) Fluorescence spectra of Singlet Oxygen Sensor Green in the suspension of **Zr-MOF-P** with and without illumination (λ_ex_ = 480 nm). (**c**) UV–Vis spectra of nitrotetrazolium blue chloride in the suspension of **Zr-MOF-P** with and without illumination. (**d**) UV–Vis spectra of coumarin-3-carboxylic acid in the suspension of **Zr-MOF-P** with and without illumination.

To further exclude the participation of ROS, electron paramagnetic resonance (EPR) tests were performed by adopting 2,2,6,6-tetramethylpiperidine (TEMP) and 5,5-dimethyl-1-pryyoline-Noxide (DMPO) as trappers. As shown in Figure 5a, no EPR signal was detected under white light irradiation, which means that no ^1^O_2_, O_2_^−•^, or ∙OH was produced by the photocatalyst. Moreover, the probe molecules Singlet Oxygen Sensor Green, nitrotetrazolium blue chloride, and coumarin-3-carboxylic acid for ^1^O_2_, O_2_^−•^, and ∙OH were added to the suspension of **Zr-MOF-P** under white light irradiation; the results still showed that no ROS was produced (Figure 5b–d). Based on the above mechanism research experiments, the possibility of ROS participating in the reaction was ruled out; sulfide was directly activated by **Zr-MOF-P,** and then reacted with ^3^O_2_.

**Figure 6:**
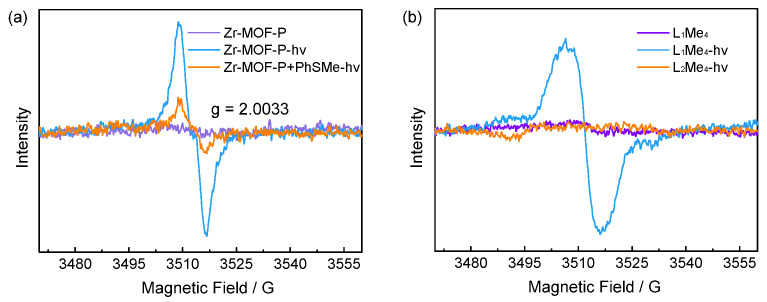
(**a**) EPR spectra of **Zr-MOF-P** under different conditions. (**b**) EPR spectra of **L_1_Me_4_** and **L_2_Me_4_** under different conditions.

**Scheme 2:**
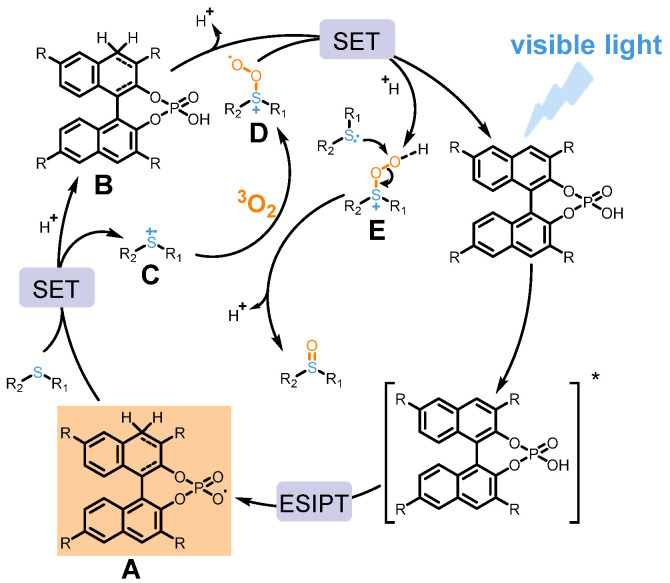
Proposed mechanism of **Zr-MOF-P** for photocatalytic oxidation of sulfides. * excited state.

The active site of **Zr-MOF-P** was found through another controlled experiment. **L_1_Me_4_** showed fairly good activity towards the reaction with a yield of 99% (Table 2, entry 9), while the phosphate-hydroxyl-protected **L_2_Me_4_** almost had no catalytic activity with a yield as low as 2% (Table 2, entry 16). EPR spectra showed a single and unstructured signal of **Zr-MOF-P** with a g value of 2.0033 after illumination, and the solid **L_1_Me_4_** also had the same signal while **L_2_Me_4_** did not, demonstrating the existence of photo-induced oxygen radicals in **Zr-MOF-P** and **L_1_Me_4_** [60]. Moreover, the EPR signal of **Zr-MOF-P** after illumination was significantly decreased after the addition of thioanisole, which indicated an electron transfer process between thioanisole and the photo-induced oxygen radical (Figure 6). Therefore, a proposed mechanism was shown in Scheme 2. The ligand was firstly excited by visible light, followed with an ESIPT process, producing the oxygen radical **A**. The photo-induced oxygen radical takes an electron from sulfide, generating the reduced ligand **B** and sulfide radical cation **C**. Then **C** reacts with ^3^O_2_, which converts into the persulfoxide radical **D**. The reduced ligand **B** donates an electron to **D**, which is recovered, and **D** is transformed into persulfoxide **E**. Finally, **E** reacts with another sulfide molecule and two molecules of sulfoxide are produced.

### 2.4. Substrate Compatibility and Recyclability

**Scheme 3:**
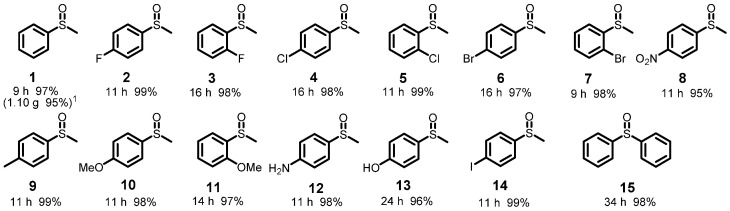
Photocatalytic oxidation of various sulfides. Conditions: sulfides (0.1 mmol), photocatalyst (4 mg), O_2_ (1 atm), solvent (2 mL), white LED (5 W), and room temperature; the yields and products were determined by GC (anisole as internal standard) and GC-MS. ^1^ Isolated yield of gram-scale experiment.

Encouraged by the unusual photocatalytic mechanism of **Zr-MOF-P**, various sulfides with different substituents were employed in the reaction (Scheme 3). Methylphenyl sulfide derivatives with halogen at the *ortho*- or *para*- positions of phenyl rings were all completely transformed into corresponding sulfoxides, and the conversion of *ortho*-substituted sulfide were slower than the *para*-substituted sulfide due to the steric hindrance (**2**–**7**). Other substituents such as nitro (**8**), methyl (**9**), and methoxy (**10**, **11**) were all tolerated in the reaction. Without the participation of ROS, amino (**12**)- and hydroxy (**13**)-substituted sulfides could be oxidized to sulfoxides and no side reaction was observed. The photosensitive iodine was also well tolerated, and almost quantitatively corresponding sulfoxide was obtained (**14**). Moreover, even the diphenyl sulfide which was difficult to be oxidized by most photocatalysts could be successfully transformed into sulfoxide (**15**) with excellent yields. All of the above results indicated that the avoidance of producing ROS would endow **Zr-MOF-P** with high substrate compatibility and selectivity.

**Figure 7:**
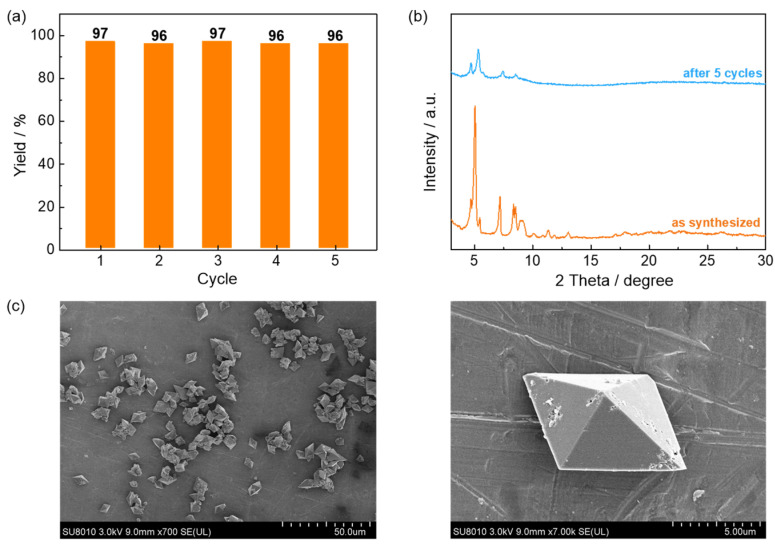
(**a**) The yield of methylphenyl sulfoxide in five cycles with **Zr-MOF-P** as photocatalyst. (**b**) PXRD patterns of **Zr-MOF-P** before and after five cycles. (**c**) SEM images of **Zr-MOF-P** after five cycles.

As a heterogenous photocatalyst, recyclability is an advantage compared with homogeneous catalysts. **Zr-MOF-P** can be easily separated through centrifugation when the reaction finished, and it can be directly used for subsequent runs without additional processing. The photocatalytic activity of **Zr-MOF-P** shows no noticeable change after five cycles of the photo-oxidation of thioanisole (Figure 7a). PXRD spectra and SEM images of the recycled photocatalyst also show little change compared with the pristine **Zr-MOF-P** (Figure 7b,c). Therefore, a photocatalyst with high stability and recyclability for the photo-oxidation of sulfides has been constructed.

## 3. Materials and Methods

### 3.1. Instruments

Powder X-ray diffraction (PXRD) was carried out with a PANalytical X’Pert3 Powder-17005730 X-ray Powder Diffractometer equipped with two Cu anodes (λ_1_ = 1.540598 Å, λ_2_ = 1.544426 Å, ratio K_-α2_/K_-α1_ = 0.5) at 40 kV and 40 mA. Thermogravimetric analysis (TGA) was performed using a TA Discovery SDT 650 heated from room temperature to 800 °C under N_2_ atmosphere at the heating rate of 10 °C·min^−1^. Scanning electron microscopy (SEM) images were obtained using a Hitachi SU-8010 microscope (Tokyo, Japan). UV–Vis diffuse reflectance spectra were obtained on a Shimadzu UV-2600i (Kyoto, Japan) spectrophotometer equipped with an integrated sphere and a white standard of BaSO_4_ was used as a reference. UV–Vis spectra were obtained on a Shimadzu UV-2600i spectrophotometer. Fluorescence spectra and quantum yield were obtained on an Edinburgh Instruments FLS1000 fluorescence spectrophotometer (Livingston, UK). Nuclear magnetic resonance (NMR) data were collected on a Bruker Avance III 500 spectrometer (Berlin, Germany). HRMS was recorded on an Agilent G6545 Q-TOF (Santa Clara, CA, USA). Electrochemical characterizations were carried out with a CH Instruments CHI660E workstation (Shanghai, China). The photocatalytic reactions were performed in a PerfectLight PCX50C photoreactor (Beijing, China) with 5 W white light LED. Gas chromatographic (GC) analyses were performed using a Shimadzu 2010 gas chromatograph (Kyoto, Japan) equipped with an HP-5MS capillary column (30 m × 0.25 mm × 0.25 μm) and a flame ionization detector. Gas chromatography–mass spectrometry (GC-MS) was recorded on a Waters GCT Premier mass spectrometer (Milford, MA, USA). Electron paramagnetic resonance (EPR) measurements were carried out on a Bruker model A300 spectrometer (Berlin, Germany).

### 3.2. Synthesis

All the reagents in experiments are commercially available and used without further purification. **L_1_H_4_** was synthesized from BINOL according to literature [7], and we optimized the reported synthesis route. Considering **I** was prepared via several protection and deprotection reactions from 6,6′-dibromo-3,3′-diiodo-1,1′-binaphthyl-2,2′-diol in literature, we tried to synthesize **I** directly through a Suzuki-Miyaura coupling reaction between 6,6′-dibromo-3,3′-diiodo-1,1′-binaphthyl-2,2′-diol and 4-(methoxycarbonyl) benzeneboronic acid.

The synthesis of **I** is as follows: 6,6′-dibromo-3,3′-diiodo-1,1′-binaphthyl-2,2′-diol (2.00 g, 2.87 mmol), 4-(methoxycarbonyl)benzeneboronic acid (5.17 g, 28.74 mmol), Pd(OAc)_2_ (129 mg, 0.57 mmol), Na_2_CO_3_ (2.13 g, 20.09 mmol), DMF (32 mL), and H_2_O (32 mL) were added into a 350 mL Schlenck tube under Ar atmosphere. The reaction was stirred for 24 h in a 60 °C oil bath. After the reaction finished, it was cooled to room temperature, the mixture was extracted with CH_2_Cl_2_, and the organic phase was washed with H_2_O three times. Then, the organic phase was dried over anhydrous Na_2_SO_4_, and the solvent was filtered and concentrated. Crude product was purified by column chromatography on silica gel (2/1 petroleum ether/ethyl acetate, *R*_f_ = 0.55) to afford 1.44 g (1.75 mmol, 61% yield) of **I** as a white solid.

The synthesis of **L_2_Me_4_** is as follows: **L_1_Me_4_** (50.0 mg, 0.056 mmol), dimethyl sulfate (14.3 mg, 10.7 μL, 0.113 mmol), NaHCO_3_ (10.4 mg, 0.124 mmol), and *N*,*N*-Dimethylacetamide (0.7 mL) were added into a 10 mL Schlenck tube under Ar atmosphere. The reaction was stirred for 24 h at room temperature. After the reaction finished, H_2_O (2 mL) was added to quench the reaction, the mixture was extracted with CH_2_Cl_2_, and the organic phase was washed with H_2_O three times. Then, the organic phase was dried over anhydrous Na_2_SO_4_, and the solvent was filtered and concentrated. Crude product was purified by column chromatography on silica gel (2/1 petroleum ether/ethyl acetate, *R*_f_ = 0.30) to afford 38.6 mg (0.043 mmol, 76% yield) of **L_2_Me_4_** as a white solid. ^1^H NMR (500 MHz, CDCl_3_) δ 8.26–8.16 (m, 12H), 7.84 (d, *J* = 7.7 Hz, 4H), 7.80 (t, *J* = 8.1 Hz, 4H), 7.69–7.65 (m, 2H), 7.56 (d, *J* = 8.8 Hz, 1H), 7.49 (d, *J* = 8.9 Hz, 1H), 4.01–3.91 (m, 12H), 3.14 (d, *J* = 9.9 Hz, 3H) (Figure S2). ^13^C NMR (126 MHz, CDCl_3_) δ 167.00, 166.89, 166.86, 166.74, 144.51, 144.44, 141.10, 141.05, 137.99, 137.95, 133.95, 133.11, 132.34, 131.95, 131.90, 131.86, 131.71, 131.67, 130.35, 130.34, 130.03, 130.00, 129.89, 129.77, 129.69, 129.63, 129.47, 129.43, 127.79, 127.27, 127.23, 126.91, 126.84, 126.69, 126.64, 122.55, 122.51, 77.29, 77.03, 76.78, 55.13, 52.35, 52.26, 52.25, 52.21 (Figure S3). HRMS (ESI): [M + H]^+^ Calcd for C_53_H_40_O_12_P^+^ 899.2252; Found 899.2252.

The synthesis of **Zr-MOF-P** is as follows: **L_1_H_4_** (200 mg, 0.242 mmol), ZrCl_4_ (169 mg, 0.0.725 mmol), anhydrous formic acid (10 mL), and trifluoroacetic acid (2 mL) were added in DMF (40 mL). After 10 min of ultrasonic vibration, the mixture was heated in a 100 mL Teflon-sealed autoclave at 120 °C for 3 days. Then, the mixture was cooled to room temperature, light yellow powders (310 mg) were collected through centrifugation, and washed with DMF. Because the removing of solvent molecules from MOF channels will distort the framework, **Zr-MOF-P** was dipped in DMF and was collected through suction filtration before use.

### 3.3. Electrochemical Characterization

Electrochemical characterizations were carried out using a CH Instruments CHI660E workstation through a three-electrode system in 0.2 M Na_2_SO_4_ aqueous solution.

Mott–Schottky plots of **Zr-MOF-P** were measured using the photocatalyst-coated glassy carbon as working electrode, Ag/AgCl as reference electrode, and Pt plate as counter electrode at frequencies of 1000, 1500, and 2000 Hz, respectively. Preparation of the working electrode is as follows: 5 mg **Zr-MOF-P** was dispersed in 1 mL ethanol, and 10 μL 5 wt% Nafion was added as binder. Then, 20 μL of the solution was coated on the surface of the glassy carbon electrode and dried at room temperature. This process was repeated until the electrode was completely covered.

Photocurrent measurements of **Zr-MOF-P** were measured using the photocatalyst-coated Pt plate as working electrode, Ag/AgCl as reference electrode, and Pt plate as counter electrode, and a 40 W White light LED was used as light source. Preparation of the working electrode is as follows: 5 mg **Zr-MOF-P** was dispersed in 1 mL ethanol, and 10 μL 5 wt% Nafion was added as binder. Then, 50 μL of the solution was coated on the Pt plate and dried at room temperature. This process was repeated until 1 cm^2^ of the Pt plate was completely covered.

### 3.4. Photocatalytic Reaction

The photocatalytic reactions were performed on a PerfectLight PCX50C photoreactor (Beijing, China) equipped with 5 W white LEDs. In addition, the reaction was carried out at 25 °C by circulating refrigeration equipment. For the photo-oxidation of sulfides to sulfoxides, 4 mg photocatalyst, 0.1 mmol substrate, and 2 mL solvent were added into a 10 mL Schlenck tube under O_2_ atmosphere. The reaction mixture was magnetically stirred at 150 rpm and illuminated with 5 W white LEDs. After the reaction finished, 20 μL of anisole was added as the internal standard and stirred for 10 min. Then, the photocatalyst was separated through centrifugation and washed with solvent. The products were analyzed by GC and GC-MS.

For gram-scale reaction, thioanisol (8.86 mmol, 1.10 g), TFEA (100 mL), and **Zr-MOF-P** (20 mg) were stirred at room temperature for 7 days in oxygen atmosphere (1 atm) under the irradiation of white LEDs. After the reaction finished, photocatalyst was separated through centrifugation and washed with ethyl acetate several times. The combined organic phase was concentrated over a rotary evaporator, and **1** (1.18 g, 95%) was obtained through column chromatography as a colorless oil.

### 3.5. EPR Measurements

EPR spectra were obtained on a Bruker model A300 spectrometer (Berlin, Germany) at room temperature. The spectrometer parameters are shown as follows: sweep width, 100 G; center field, 3510.890 G; microwave bridge frequency, 9.839 GHz; power, 20.37 mW; modulation frequency, 100 kHz; modulation amplitude, 1 G; conversion time, 42.00 s; sweep time 42.00 s; receiver gain, 2.00 × 10^4^. The preparation of the liquid samples was similar to the photocatalyst reaction. The signal after irradiation was measured after 5 min of irradiation with a 50 W Xe lamp with stirring, and the mixture was transferred to 3 mm diameter glass tubes as soon as possible to record the signals. Furthermore, for solid samples, about 2 mg of target compound was put into a 3 mm diameter glass tube, and the signal after irradiation was also measured after 5 min of irradiation with a 50 W Xe lamp.

### 3.6. ROS Detection with Probe Molecules

^1^O_2_ detection: **Zr-MOF-P** (2 mg) and TFEA (1.5 mL) containing Singlet Oxygen Sensor Green (10 μM) were added into a 10 mL Schlenck tube under air atmosphere. The reaction mixture was magnetically stirred for 30 min in the dark before illuminated with white LEDs for 1 h. After the reaction finished, photocatalyst was separated through centrifugation and the supernatant was examined with fluorescence spectrophotometer. The result was compared with the blank group and unilluminated control group, showing that no ^1^O_2_ produced.

O_2_^−•^ detection: **Zr-MOF-P** (2 mg) and TFEA (1.5 mL) containing nitrotetrazolium blue chloride (0.1 mM) were added into a 10 mL Schlenck tube under air atmosphere. The reaction mixture was magnetically stirred for 30 min in the dark before illuminated with white LEDs for 2 h. After the reaction finished, photocatalyst was separated through centrifugation and the supernatant was examined with UV–Vis spectrophotometer. The result was compared with the unilluminated control group, showing that no O_2_^−•^ produced.

·OH detection: **Zr-MOF-P** (2 mg) and TFEA (1.5 mL) containing coumarin-3-carboxylic acid (0.1 mM) were added into a 10 mL Schlenck tube under air atmosphere. The reaction mixture was magnetically stirred for 30 min in the dark before illuminated with white LEDs for 2 h. After the reaction finished, photocatalyst was separated through centrifugation and the supernatant was examined with UV–Vis spectrophotometer. The result was compared with the unilluminated control group, showing that no ·OH produced.

## 4. Conclusions

A photocatalyst **Zr-MOF-P** based on a BINOL-derived phosphoric acid ligand for the selective oxidation of sulfides under white light irradiation was prepared. Comprehensive mechanistic studies indicated that **Zr-MOF-P** had appropriate photo-electrochemical properties for this reaction, and the ESIPT process produced the reactive oxygen radical, which would take an electron from the sulfides. Thus, the sulfides were activated and, subsequently, react with ground state oxygen, producing sulfoxides. The unique mechanism without the participation of ROS ensured the high selectivity and substrate compatibility of the reaction. Moreover, as a heterogeneous photocatalyst, **Zr-MOF-P** had sufficient stability, as it can be easily separated and re-used at least five times without any noticeable change in reactivity. This study demonstrates that phosphoric acids with a large conjugate structure can be used as photocatalysts, and they might have potential applications in more kinds of photocatalytic reactions. Further applications for **Zr-MOF-P** are under study in our group.

## Figures and Tables

**Table 1:** Photo-oxidation of thioanisol ^1^.

Entry	Solvent	Change in Other Conditions	Yield ^2^/%
1	MeCN	none	19
2	MeOH	none	58
3	EtOH	none	74
4	EtOAc	none	trace
5	CF_3_CH_2_OH	none	97 (95) ^3^
6	CHCl_3_	none	56
7	DMF	none	3
8	CF_3_CH_2_OH	no photocatalyst	1
9	CF_3_CH_2_OH	dark	n.d. ^4^
10	CF_3_CH_2_OH	N_2_ atmosphere	n.d. ^4^

^1^ Conditions: thioanisol (0.1 mmol), **Zr-MOF-P** (4 mg), O_2_ (1 atm), solvent (2 mL), white LED (5 W), room temperature, 9 h. ^2^ Determined by gas chromatography (GC) (anisole as internal standard) and GC-MS. ^3^ Isolated yield of gram-scale experiment. ^4^ n.d. = not detected.

**Table 2:** Quenching experiments ^1^.

Entry	Photocatalyst	Additive	Yield ^2^/%
1	**Zr-MOF-P**	none	97
2	**Zr-MOF-P**	DABCO	95
3	**Zr-MOF-P**	BQ	96
4	**Zr-MOF-P**	*i*-PrOH	94
5	**Zr-MOF-P**	catalase ^3^	93
6	**Zr-MOF-P**	HQ	10
7	**Zr-MOF-P**	CuSO_4_	18
8	**Zr-MOF-P**	DMB	78
9 ^4^	**L_1_Me_4_**	none	99
10 ^4^	**L_1_Me_4_**	DABCO	91
11 ^4^	**L_1_Me_4_**	BQ	98
12 ^4^	**L_1_Me_4_**	*i*-PrOH	92
13 ^4^	**L_1_Me_4_**	HQ	11
14 ^4^	**L_1_Me_4_**	CuSO_4_	26
15 ^4^	**L_1_Me_4_**	DMB	15
16 ^4^	**L_2_Me_4_**	none	2

^1^ Conditions: thioanisol (0.1 mmol), photocatalyst (4 mg), scavengers (0.2 mmol), O_2_ (1 atm), solvent (2 mL), white LED (5 W), room temperature, 9 h. ^2^ Determined by GC, anisole as internal standard. ^3^ 0.1 g of catalase (>200,000 unit/g) ^4^ 4 h.

## Data Availability

CCDC 2218003 contains the supplementary crystallographic data of **Zr-MOF-P**: these data can be obtained free of charge through www.ccdc.cam.ac.uk/data_request/cif (accessed on 26 November 2022), or by emailing data_request@ccdc.cam.ac.uk, or by contacting The Cambridge Crystallographic Data Centre, 12 Union Road, Cambridge CB2 1EZ, UK; fax: +44-1223-336033.

## Supplementary Materials

The following supporting information can be downloaded at: https://www.mdpi.com/article/10.3390/ijms232416121/s1.
