# Supplementary material for: Zr-Based Metal−Organic Frameworks with Phosphoric Acids for the Photo-Oxidation of Sulfides
Source: Int J Mol Sci. 2022 Dec 17;23(24):16121. doi: 10.3390/ijms232416121 (PMC9784696; doi:10.3390/ijms232416121)
Supplement: Supplementary file 1 [file ijms-23-16121-s001.zip › ijms-2090910-supplementary.pdf]

SUPPLEMENTARY MATERIALS

Zr-Based Metal−Organic Frameworks with Phosphoric Acids for the Photo-oxidation of Sulfides

Zhenghua Zhao [1,2], Mingjie Liu [1,2], Kai Zhou [1,2], Hantao Gong [1,2], Zongbi Bao [1,2], Qiwei Yang [1,2], Qilong Ren [1,2], and Zhiguo Zhang [1,2]*

[1] Key Laboratory of Biomass Chemical Engineering of Ministry of Education, College of Chemical and Biological Engineering, Zhejiang University, Hangzhou 310058, China
[2] Institute of Zhejiang University−Quzhou, Quzhou 324000, China
* Correspondence: zhiguo.zhang@zju.edu.cn

Table S1. Crystal data and structural refinement for **Zr-MOF-P**.

Compound	**Zr-MOF-P**
empirical formula	$C_{96}H_{66}O_{40}P_2Zr_6$
formula weight	2468.74
crystal system	monoclinic
space group	*C 2/m*
a / Å	21.112(5)
b / Å	38.991(6)
c / Å	19.209(4)
α / degree	90
β / degree	120.902(12)
γ / degree	90
V / Å^3	13568(5)
Z	2
F(000)	2464
θ range collected / degree	2.332 to 53.241
limiting indices	$-24 \leq h \leq 20$
	$-45 \leq k \leq 46$
	$-22 \leq l \leq 22$
reflections collected / unique	65806 / 11860
data / restraints / parameters	11860 / 205 / 287
R (int)	0.2171
goodness-of-fit on F^2	1.006
Final R indices ([$I > 2\sigma(I)$])	R_1 = 0.1521 wR_2 = 0.4007
R indices (all data)	R_1 = 0.2755 wR_2 = 0.4810

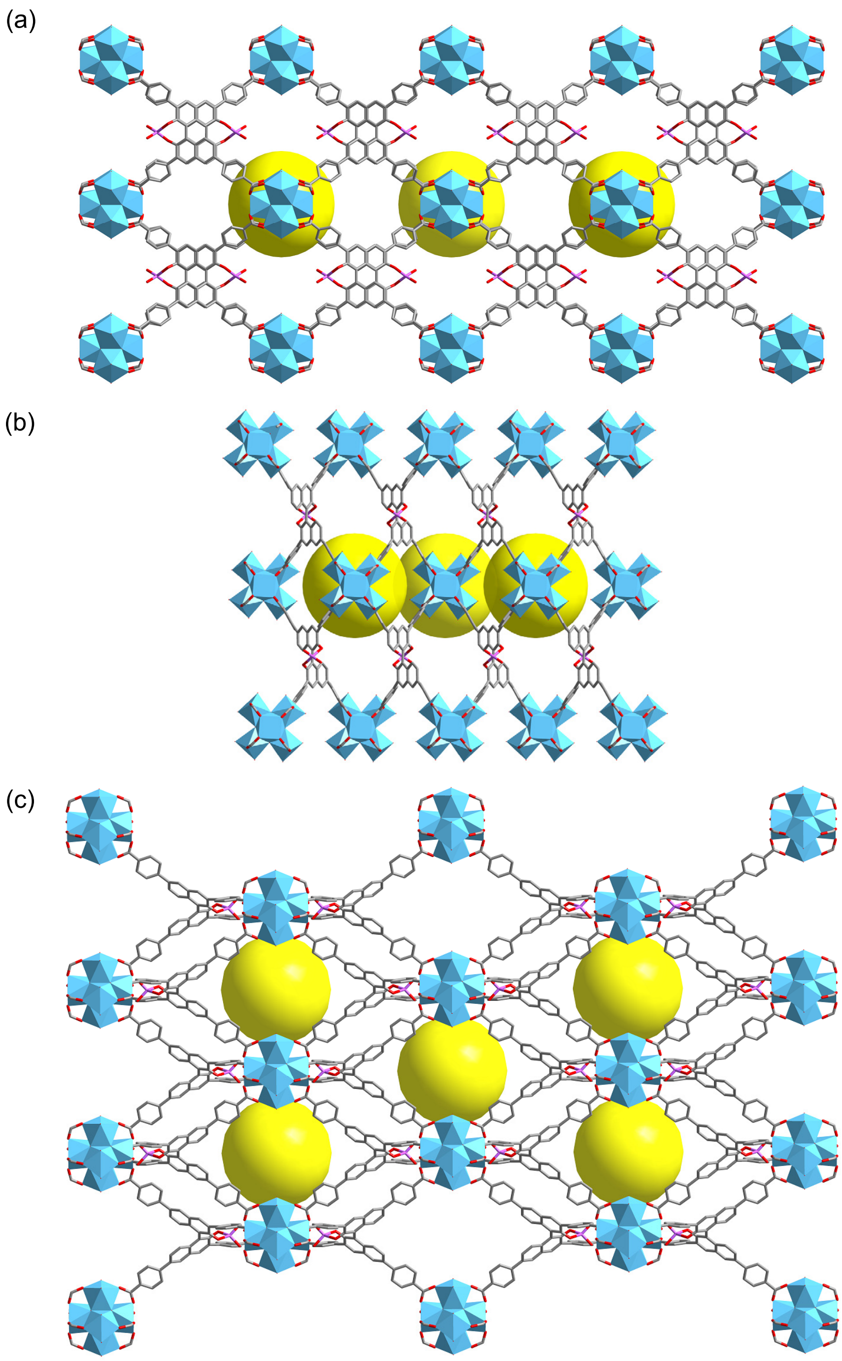

Figure S1. Crystal structure of **Zr-MOF-P**, viewed along a axis (a), b axis (b) and c axis (c).

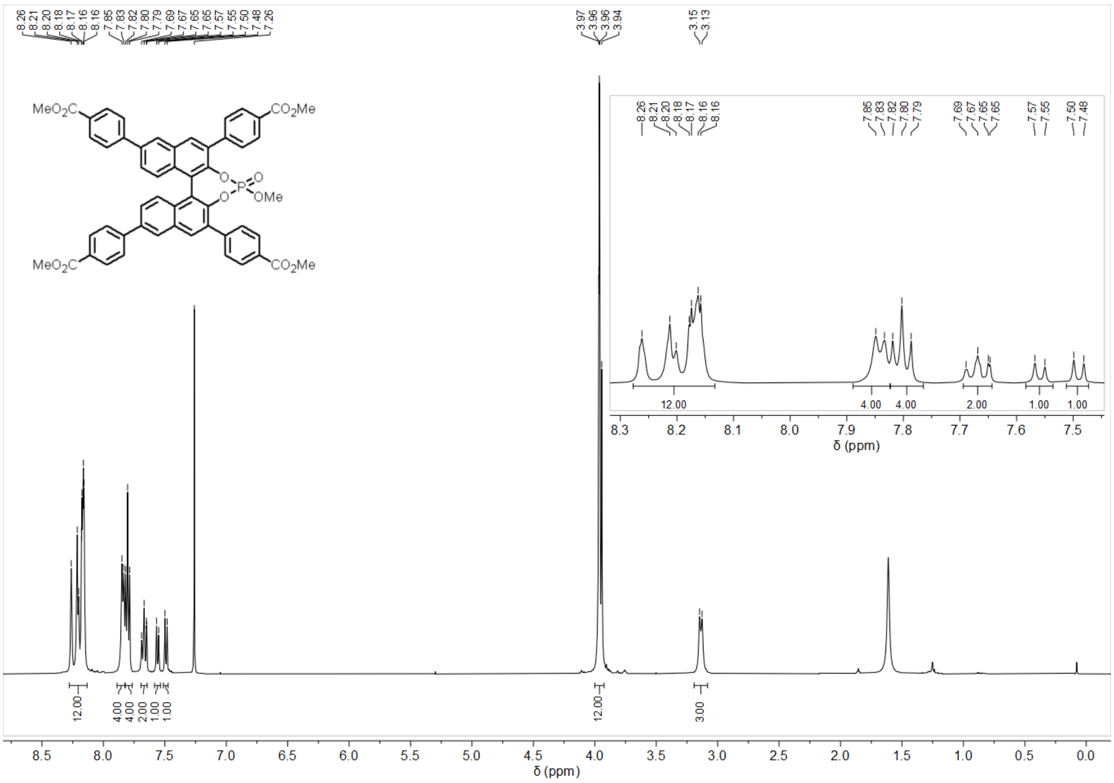

Figure S2. ^{1}H NMR of **L2Me4** (500 MHz, CDCl3, r.t.)

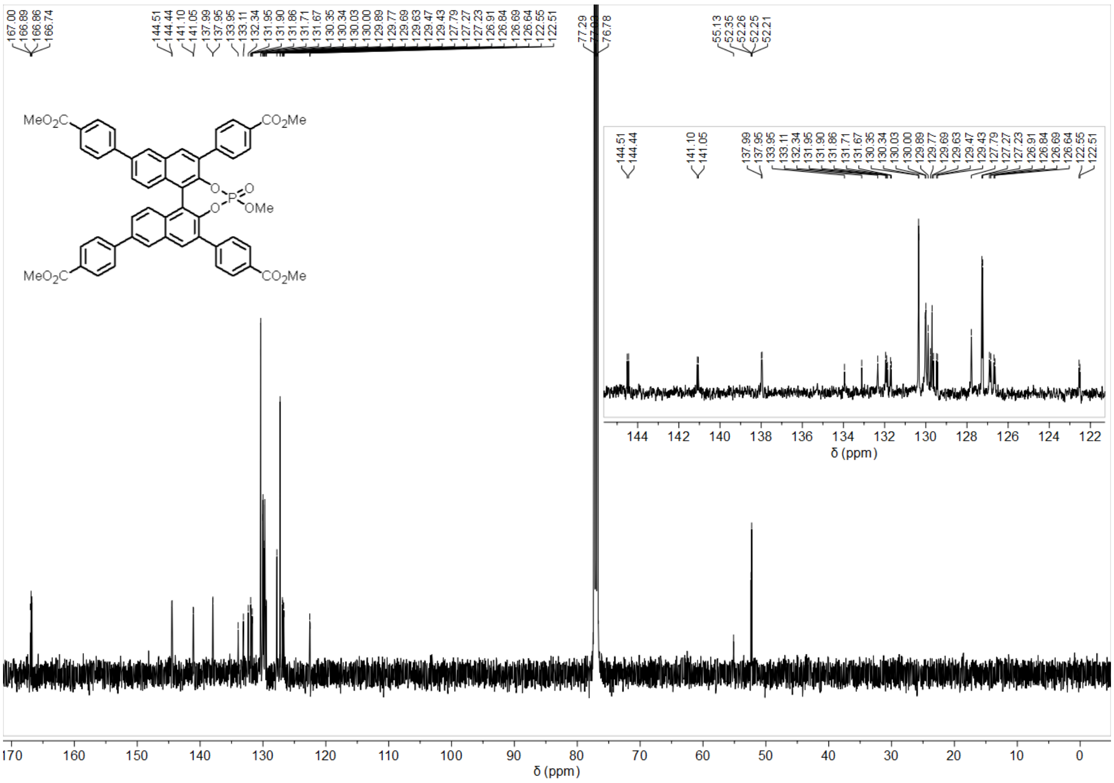

Figure S3. ^{13}C NMR of **L2Me4** (126 MHz, CDCl3, r.t.)
